# Retinol and cholecalciferol affect buserelin-induced estrous in anestrous mares

**DOI:** 10.1007/s11250-025-04369-9

**Published:** 2025-03-17

**Authors:** Syed S. U. H. Bukhari, Sundas Urooj

**Affiliations:** 1 Department of Veterinary Clinical Sciences, Jockey Club College of Veterinary Medicine and Life Sciences, City University of Hong Kong, Hong Kong, 999077 SAR China; 2 Centre for Animal Health and Welfare, Jockey Club College of Veterinary Medicine and Life Sciences, City University of Hong Kong, Hong Kong, 999077 SAR China; 3 Department of Theriogenology, Faculty of Veterinary Science, University of Agriculture, Faisalabad, 38000 Pakistan; 4 Department of Clinical Medicine and Surgery, Faculty of Veterinary Science, University of Agriculture, Faisalabad, 38000 Pakistan

**Keywords:** Anestrous mare, Equine reproduction, Estrogen, Estrous induction, Follicle development, Progesterone

## Abstract

In winter anestrous, prolonged melatonin secretion inhibits gonadotropin-releasing hormone (GnRH). However, synthetic GnRH analogues such as buserelin can stimulate follicular development. We aimed to investigate clinical relationship between retinol, cholecalciferol, and buserelin for inducing estrous in anestrous mares (*Equus caballus*). We used a total of twenty-one anestrous mares, randomly divided into three groups of seven animals. Group A received retinol, cholecalciferol, and buserelin; group B received buserelin; and group C was control. Groups A, B, and C had 71.42% (*n* = 5; 95% CI: 26.30%-96.56%), 28.57% (*n* = 2; 95% CI: 16.56%-73.70%), and 0% mares in estrous, respectively. A significantly greater number of group A mares exhibited estrous (*P* = 0.005) and higher ovarian follicular size (*P* = 0.001) compared to group C. Serum estrogen was significantly higher in group A compared to Group B (*P* = 0.03) and C mares (*P* = 0.001). In multiple correspondence analysis (MCA) factor map, treatment with retinol, cholecalciferol, and buserelin was clustered with estrous mares having serum estrogen levels > 40 pg/ml and follicular size > 30 mm. The variance explained by the first two dimensions of MCA was 87.83%. Supplementing with retinol and cholecalciferol improved the rate of buserelin-induced estrous in anestrous mares. Further research is necessary to determine underlying mechanisms.

## Introduction

Horses (*Equus caballus*) are used worldwide for sports, entertainment, leisure, transportation, and work (Bukhari et al. 2021; Bukhari and Parkes 2023; Ahmad et al. 2025). There are approximately 58.8 million horses globally (Khadka 2015). The foundation of equine industry is horse breeding, with the aim of producing healthy and higher numbers of foals (Macleay et al. 2022). Mares are long-day breeders, where their pineal gland receives stimulation from the retina as the duration of daylight increases, suppressing melatonin release and positively affecting GnRH secretion from the hypothalamus (Polasek et al. 2017; Zafar et al. 2020). Increased GnRH secretion from the hypothalamus triggers gonadotropins release from the anterior pituitary, leading to the resumption of ovarian activity (Polasek et al. 2017; Zafar et al. 2020). In winter, prolonged secretion of melatonin due to long periods of darkness inhibits secretion of GnRH, resulting in decreased secretion of gonadotropins, luteinizing hormone (LH), and follicle-stimulating hormone (FSH) (Aurich 2011). However, synthetic GnRH analogues like deslorelin during early anestrous can stimulate follicular development, leading to mares ovulating in response to hCG (human chorionic gonadotropin) (Kwong and Klein 2019). Additionally, naltrexone, a predominantly m-receptor opioid antagonist, stimulates FSH and LH secretion in anestrous mares (Irvine 1994). Therefore, exogenous GnRH analogues such as deslorelin, naltrexone, and buserelin can induce estrous in anestrous mares (Kwong and Klein 2019; Zafar et al. 2020).

Optimal reproduction requires presence of essential vitamins, such as retinol (vitamin A) and cholecalciferol (vitamin D) (Ikeda et al. 2005; Franasiak et al. 2017). Cholecalciferol is increasingly recognized as essential for female reproduction, and optimal levels are crucial for reproductive function (Franasiak et al. 2017). Though, underlying mechanisms are inconclusive due to the intricacy of cholecalciferol metabolism and its widespread effects in regulating physiological processes in reproductive, endocrine, and neurological systems that control reproduction (Xu et al. 2021). However, beliefs regarding its effect on folliculogenesis, endometrial receptivity, and ovarian functions are well established (Franasiak et al. 2017). Furthermore, cholecalciferol regulates blood calcium levels, which is essential for cell signaling and functions. Together, cholecalciferol and calcium enhance female reproductive health and performance (Giorgi et al. 2018; Dickerson et al. 2022; Safari et al. 2022). Additionally, retinol is a micronutrient associated with efficient reproductive function (Ikeda et al. 2005). It travels to the ovarian follicles, where it works as an antioxidant and gene regulator, aiding in the development and maturation of oocytes (Ikeda et al. 2005).

We aimed to determine clinical relationship between retinol, cholecalciferol, and buserelin for inducing estrous in late-season anestrous mares. We hypothesized that supplementation with retinol and cholecalciferol could enhance the onset of estrous induced by buserelin in anestrous mares.

## Materials and methods

### Sample size estimation

The sample size was estimated using G*Power 3.1 software (Faul et al. 2007). Previous research reported that cholecalciferol is essential for follicle development and ovarian function, as it helps mediate actions of gonadotropins (Xu et al. 2021). Additionally, retinol has beneficial effects on fertility; it promotes maturation of oocyte cytoplasm and modulates gene expression of gonadotropin receptors in ovarian follicles (Ikeda et al. 2005). For the current research, we hypothesized that cholecalciferol and retinol enhance effectiveness of buserelin in promoting follicular development and inducing estrous. Therefore, the input parameters set for G*Power were alpha (α = 0.05), power (0.80), and effect size (ρ = 0.5), leading to a sample size estimation of 21 mares for the current study.

## Research area and selection of mares

The research was conducted during the months of October and November 2019, at The Remount Depot Mona, Mandi Bahauddin, Punjab, Pakistan. This region is part of the irrigated plains of Pakistan, with an elevation of 220 m above sea level (Hussain et al. 2024). The highest average monthly temperature of 48 °C occurs in June, while the lowest, at 3 °C, was recorded in January (Hussain et al. 2024). The region receives an average rainfall of 388 mm (15.3 inches) and experiences all four seasons throughout the year (Hussain et al. 2024).

**Fig. 1:**
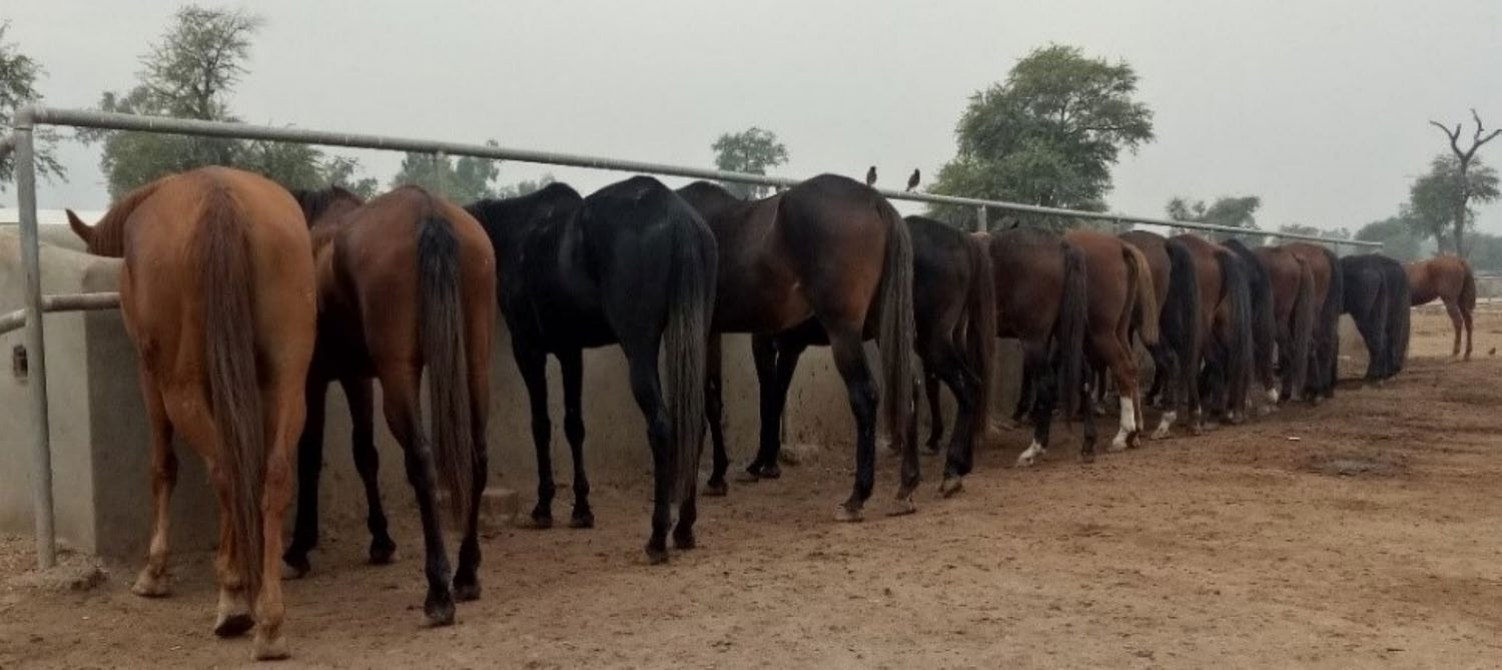
Experimental mares feeding in a paddock. Photo: Syed S. U. H. Bukhari

Informed consent was obtained from equine stud before the start of study. Twenty-one Thoroughbred anestrous mares, ranging in age from six to ten years and weighing 600–750 kg, were selected and kept in a separate paddock with the same feeding, housing, and farming practices, Fig. 1. Mares were assessed at rest by an equine veterinary surgeon for overall health. They were free from any disease and had not been prescribed any medication during the study period or for three months prior to data collection. The reproductive systems of mares were examined using rectal ultrasonography to identify anestrous animals. We used a B-mode ultrasound machine (Honda Electronics, model HS-1500) with a linear array transducer. The ultrasound examination was conducted at a frequency of 7.5 megahertz, and mares were considered anestrous if they had no or very small corpus luteum and follicles (5–10 mm) (Nie et al. 2006). This ensured that mares were anestrous, non-pregnant, and free from endometritis, pyometra, and follicular or luteal cysts.

### Experimental design

Mares were randomly divided into three groups (A, B, and C), each consisting of seven animals. In group A, first treatment group, mares were given intramuscular (IM) administration of retinol and cholecalciferol (Lucky Core Industries, Pakistan) on days one, four, and seven. This injection also included a small amount of alpha-tocopherol acetate as a preservative. On day seven, first dose of buserelin (Star Laboratories, Pakistan) was also administered IM along with retinol and cholecalciferol, and a daily dose of buserelin was continued for a maximum of eight days (day 7–14). We ceased further administration of buserelin once mares were detected in estrous within the eight-day buserelin protocol. Group B mares, second treatment group, received an eight-day buserelin protocol (days 7 to 14) via IM buserelin administration. Similar to group A mares, we ceased further administration of buserelin once mares were detected in estrous within the eight-day buserelin protocol. Group C was control, and mares were kept in the same paddock as the treatment animals. The treatment protocol and schedule for groups A, B, and C are presented in Table 1.

**Table 1:** Day-wise treatment schedule for mares in groups A, B, and C. The symbol "IU" stands for international units, "µg" refers to micrograms, and "IM" means intramuscular

Day	Group A	Group B	Group C
1	800,000 IU retinol IM + 400,000 IU cholecalciferol IM	No treatment	No treatment
4	800,000 IU retinol IM + 400,000 IU cholecalciferol IM	No treatment	No treatment
7	800,000 IU retinol IM + 400,000 IU cholecalciferol IM + 10.50 µg buserelin IM	10.50 µg buserelin IM	No treatment
8	10.50 µg buserelin IM	10.50 µg buserelin IM	No treatment
9	10.50 µg buserelin IM	10.50 µg buserelin IM	No treatment
10	10.50 µg buserelin IM	10.50 µg buserelin IM	No treatment
11	10.50 µg buserelin IM	10.50 µg buserelin IM	No treatment
12	10.50 µg buserelin IM	10.50 µg buserelin IM	No treatment
13	10.50 µg buserelin IM	10.50 µg buserelin IM	No treatment
14	10.50 µg buserelin IM	10.50 µg buserelin IM	No treatment

### Estrous detection

**Fig. 2:**
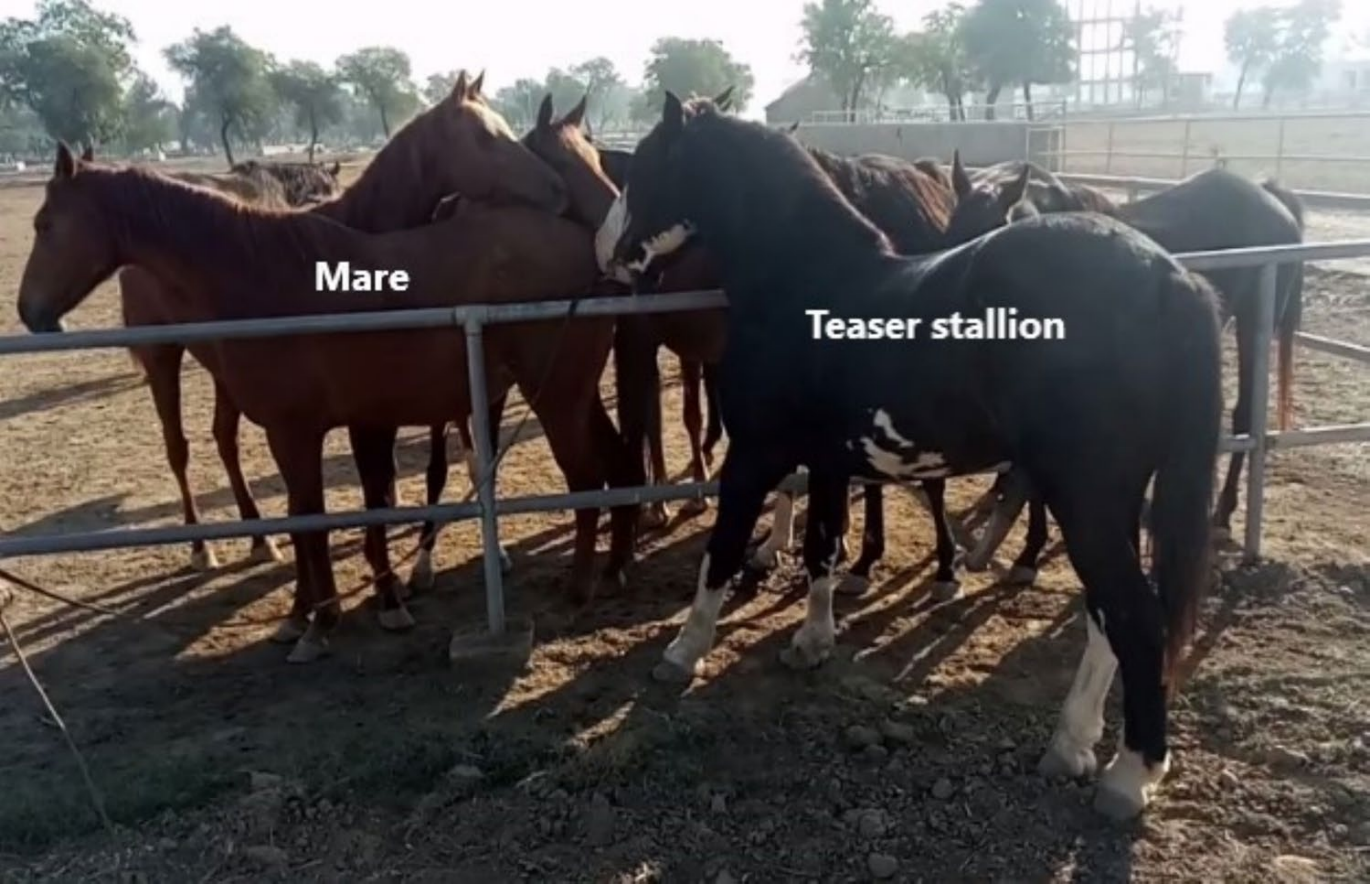
Estrous detection using a teaser stallion outside the paddock with mares inside. Photo: Syed S. U. H. Bukhari

A stallion was used as a teaser, and daily teasing started with the eight-day buserelin protocol on day seven from 0800 to 0900 in the morning, Fig. 2. The reaction of mares towards teasing was recorded using teasing score (TS) criteria (Coleman and Powell 2004), Table 2. Rectal ultrasonography was performed to confirm estrous in mares showing signs of estrous (TS two to four). One equine veterinarian (SSUHB) assessed estrous signs and performed ultrasonography to avoid confounding factors associated with multiple accessors.

**Table 2:** Criteria for identifying estrous in mares using teasing scores (TS), (Coleman and Powell 2004)

TS	Criteria
Zero	Mare shows no signs of receptivity, aggression towards stallion, kicking, and squealing
One	Mare shows no aggression towards stallion
Two	Mare shows attraction towards stallion, winking of vulva, and tail elevation
Three	Mare shows interest towards stallion, raising tail, squatting, and urination
Four	Mare shows strong interest towards stallion, moves hindquarters towards stallion, and tail elevation

### Measurement of ovarian follicular size

**Fig. 3:**
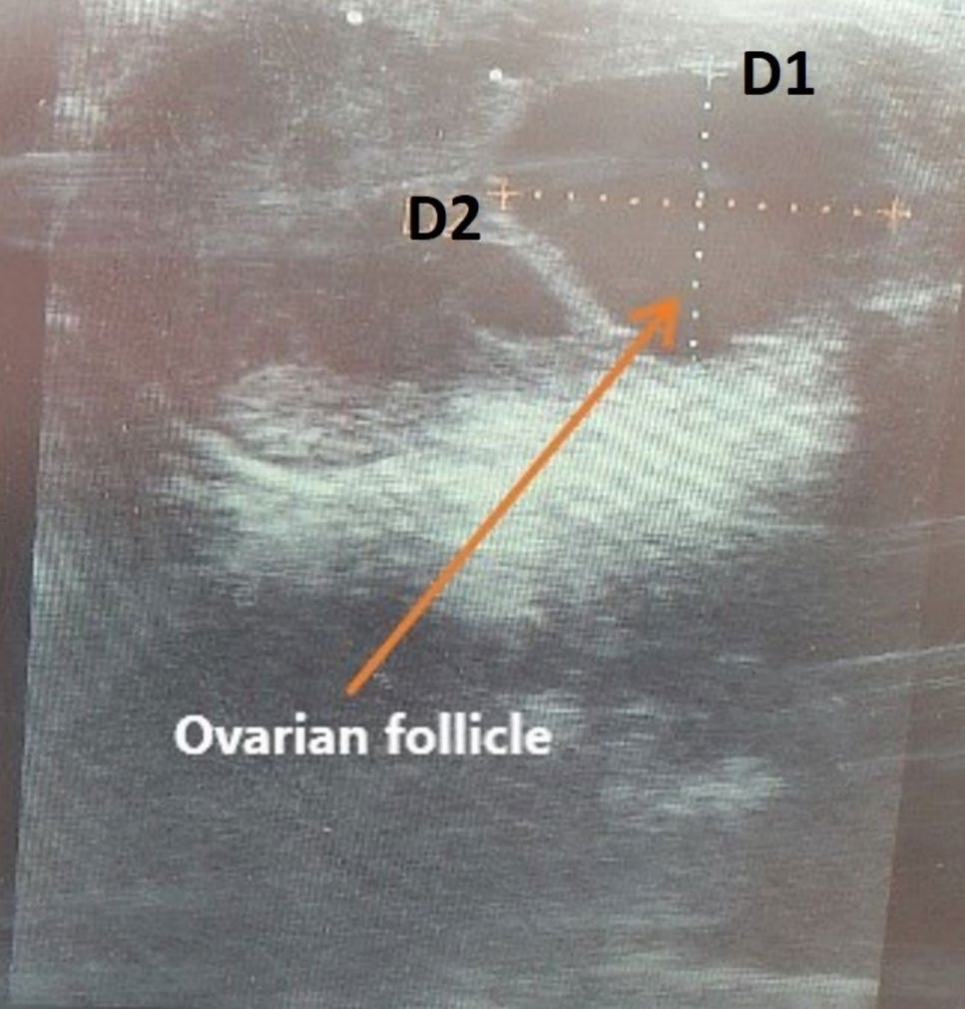
Measurement of ovarian follicle size using rectal ultrasonography. D1 and D2 represent the vertical and horizontal diameters, respectively. Image: Syed S. U. H. Bukhari

Follicular size (mm) was measured using rectal ultrasonography (Honda Electronics, model HS-1500) according to existing method (Mokhtari et al. 2016). The size of the ovarian follicles was determined by measuring their diameters in two directions, vertical (D1) and horizontal (D2). In cases where two follicles were present, both diameters (D1 and D2) of the dominant follicle were measured, and the average diameter (AD) was calculated to estimate size of the follicle, Fig. 3.

$$\text{Average diameter }(\text{AD})=\frac{\text{D}1+\text{D}2}{2}$$

### Measurement of serum progesterone and estrogen

Blood samples (twenty-one) were collected from the jugular vein, and serum was isolated to measure progesterone (ng/ml) and estrogen (pg/ml). Blood samples were taken from all experimental mares on day 14 of the treatment. Commercially available radioimmunoassay (RIA) kits (www.beckmancoulter.com) were used to quantify progesterone and estrogen levels in mare serum samples according to existing guidelines (Corona et al. 2010; Handelsman et al. 2014).

### Statistical analysis

Categorical data (exhibited estrous or not) were described as counts, percentages, and 95% confidence intervals (95% CI). Continuous variables (ovarian follicular size, serum estrogen, and progesterone levels) were presented as the median and interquartile range (boxplots). Odds ratios (OR) and Chi-Square (X^2^) tests were used to compare onset of estrous (Yes or No) among groups A, B, and C. One-way ANOVA (analysis of variance) was used to compare follicular size, serum estrogen, and progesterone levels among three groups of mares on day 14 of treatment. In this analysis, groups served as the independent variables, while follicular size, serum estrogen, and progesterone levels were the dependent variables. Tukey test was used for post-hoc comparisons for each ANOVA model. Moreover, the relation between response (exhibited estrous or not) of groups A, B, and C mares to their respective treatments, serum estrogen, and follicular size was analyzed using multiple correspondence analysis (MCA). Binary variables were created for ovarian follicular size (> 30 mm or < 30 mm) and serum estrogen level (> 40 pg/ml or < 40 pg/ml) on day 14 of treatment. This classification was based on previous studies indicating that the dominant preovulatory follicular size is typically greater than 30 mm, while serum estrogen levels exceed 40 pg/ml (Oxender et al. 1975; Gastal et al. 1999; Abo-El maaty and El-Shahat 2012). Progesterone remained a single category variable as values for all the 21 mares were similar, between 0.17–0.23 ng/ml on day 14 of the treatment. Spatially clustered variables were regarded as being similar, with spatial distance reflecting rare associations (Greenacre 2007; Alhuzali et al. 2022; Bukhari et al. 2023). Where present, an appropriate name was given to describe the cluster. The MCA factor map included treatments (for groups A, B, and C), response of mares (estrous induced or not), ovarian follicular size, serum estrogen, and progesterone levels. All statistical analyses were conducted using the open-source software RStudio version 2022.07.1–554.

## Results

### Effect of buserelin treatment alone and in combination with retinol and cholecalciferol on the onset of estrous

**Fig. 4:**
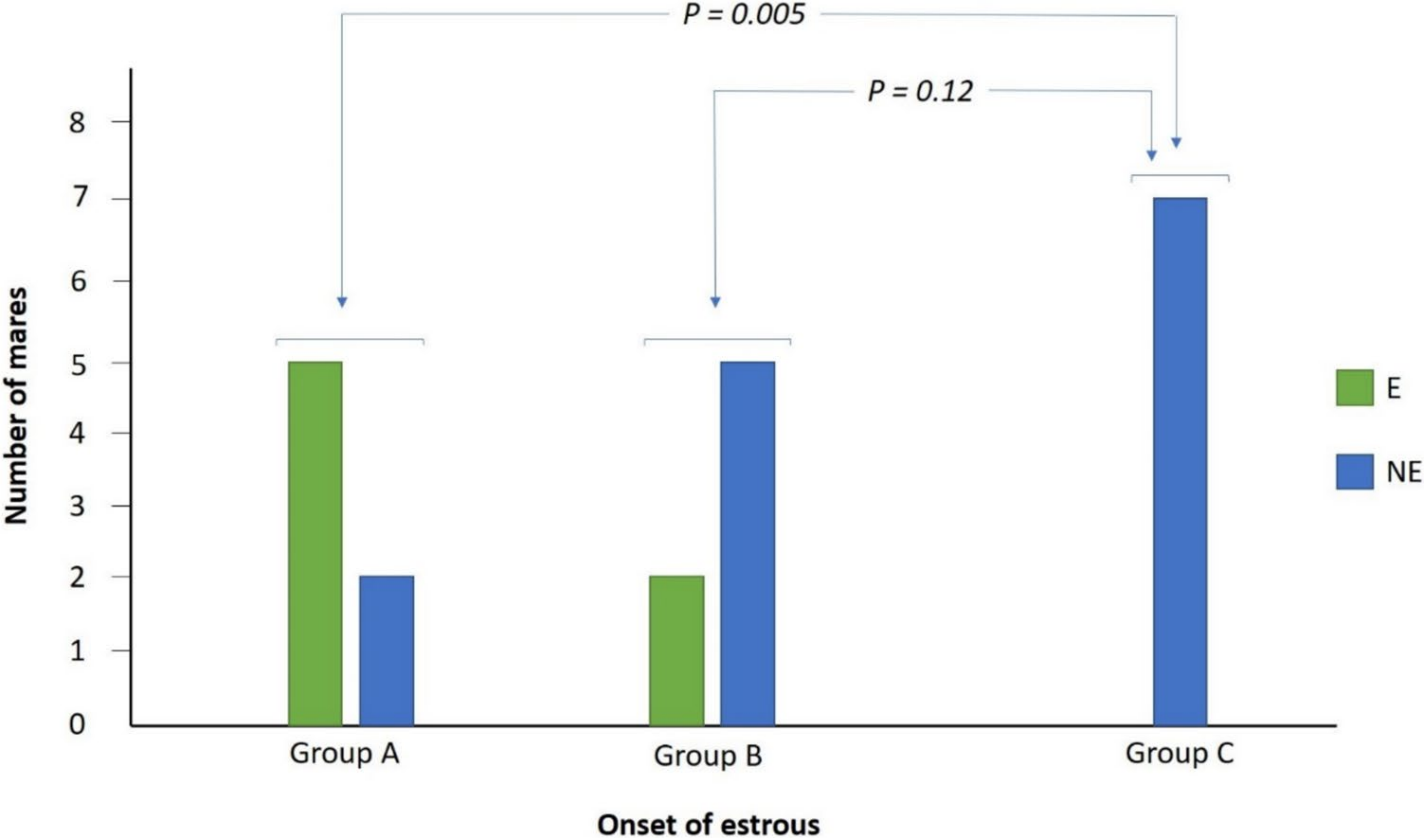
Number of mares exhibited estrous following treatment (groups A and B) and in the control group (C). Symbols "E" and "NE" represent mares that exhibited estrous and did not exhibit estrous, respectively. P-values were generated using the Chi-Square (X^2^) test for categorical variables

Groups A, B, and C had 71.43% [*n* = 5; 95% CI (26.30–96.56%)], 28.57% [*n* = 2; 95% CI (16.56–73.70%)], and zero mares in estrous, respectively. A significantly greater number of group A mares exhibited estrous compared to group C (X^2^ = 7.78, OR = 0.71, *P* = 0.005), while no significant association was found between groups B and C (X^2^ = 2.33, OR = 0.28, *P* = 0.12), Fig. 4.

### Ovarian follicular size

**Fig. 5:**
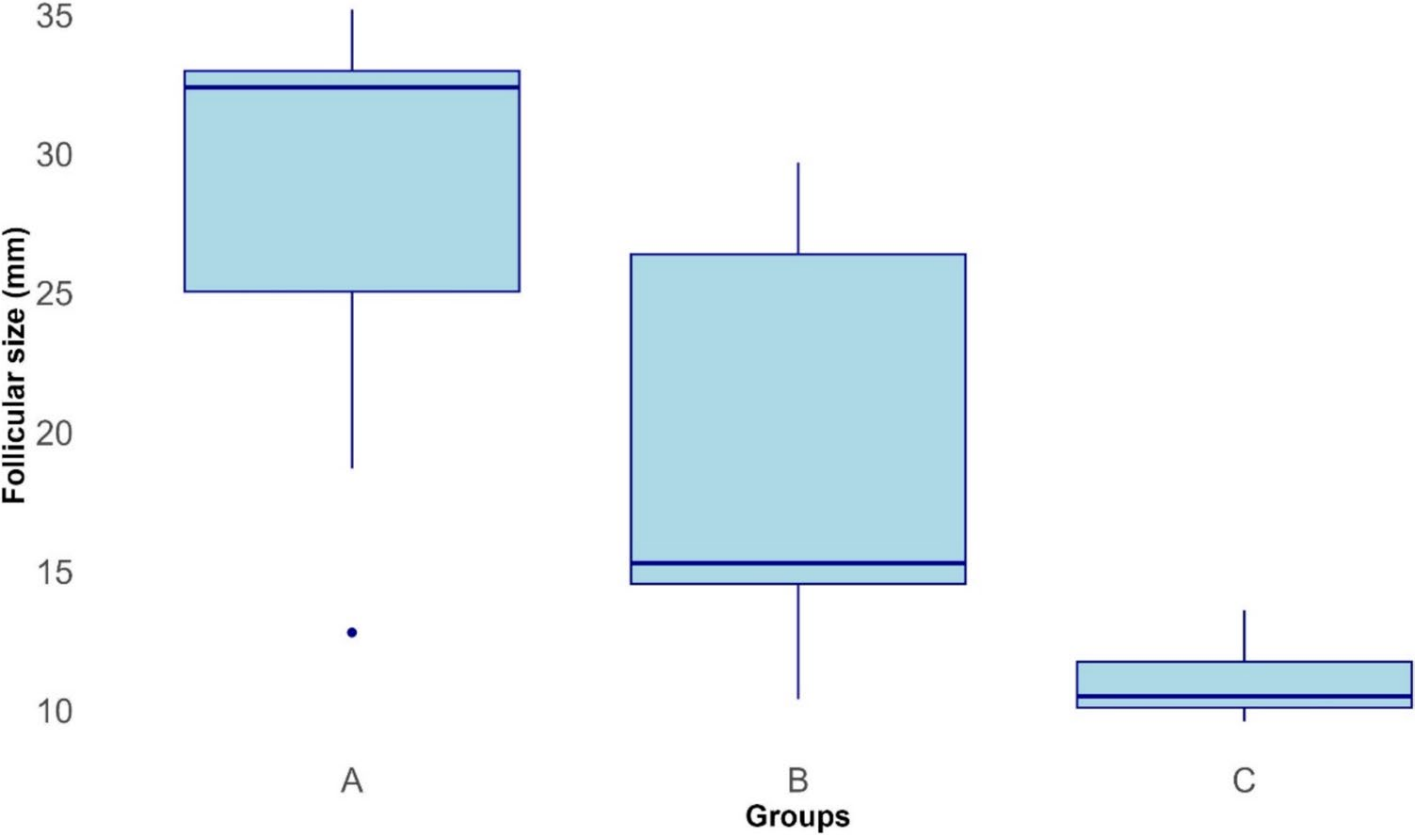
Boxplots showing ovarian follicular size (mm) of group A, B, and C mares on day 14

Median follicular sizes of mares in groups A, B, and C are shown in Fig. 5. Follicular size was significantly different between groups A and C (*P* < 0.001). However, no significant difference was observed between groups B and A, and B and C.

### Serum estrogen level

**Fig. 6:**
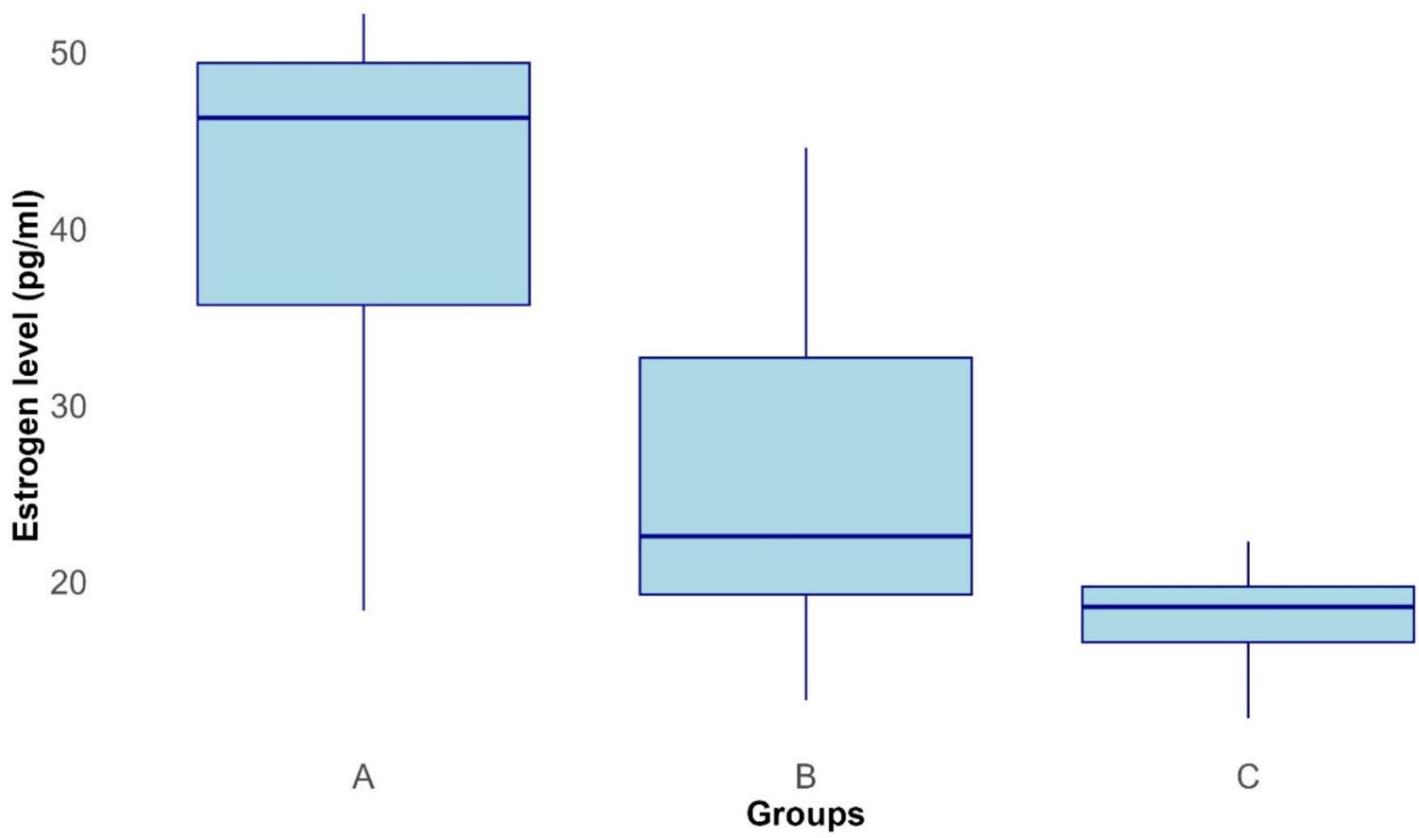
Boxplots showing serum estrogen level (pg/ml) of group A, B, and C mares on day 14

Median serum estrogen levels of mares in groups A, B, and C are shown in Fig. 6. Estrogen levels were significantly different between groups A and C (*P* = 0.001), and A and B mares (*P* = 0.03). However, no significant difference was observed between groups B and C.

### Serum progesterone level

**Fig. 7:**
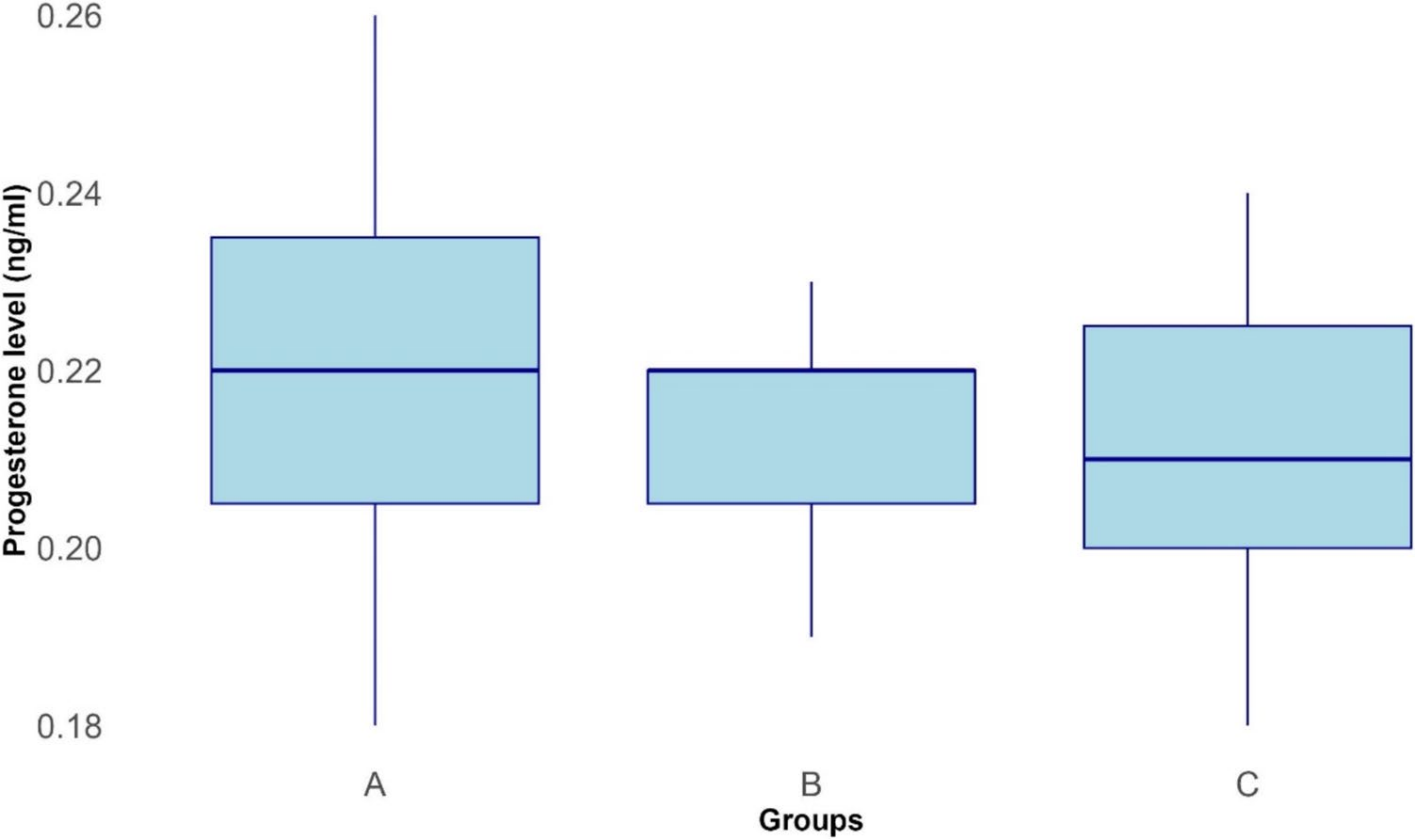
Boxplots showing serum progesterone level (ng/ml) of group A, B, and C mares on day 14

Median serum progesterone levels of mares in groups A, B, and C are shown in Fig. 7. There were no significant differences in progesterone levels between groups A and C, groups A and B, and groups B and C.

### Multiple correspondence analysis of treatments and responses

**Fig. 8:**
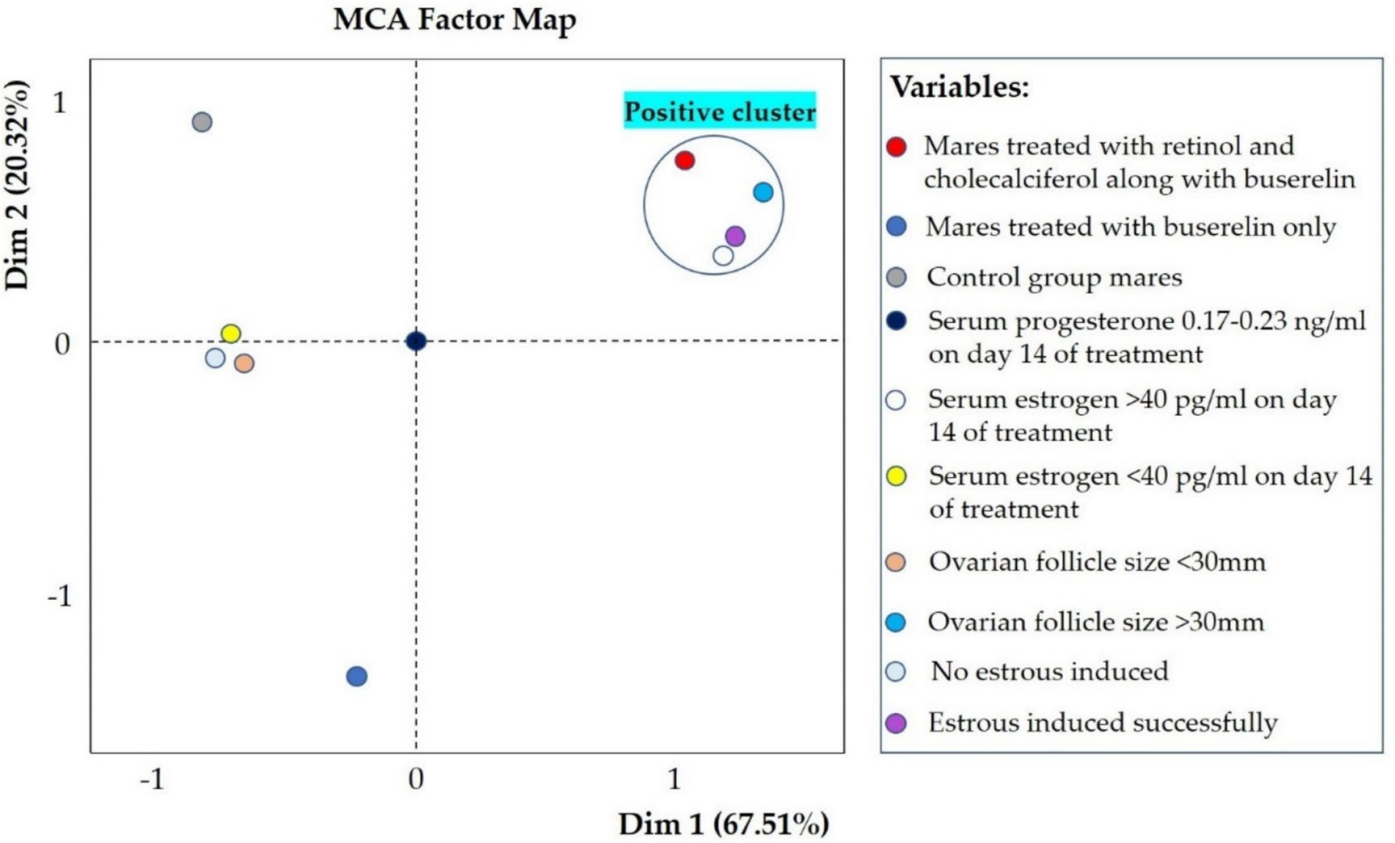
MCA factor map showing the relation among studied variables. Variance explained by first and second dimension was 67.51% and 20.32%, respectively. Colored dots represent variables, and the ‘positive cluster’ indicates mares that responded positively to treatment by exhibiting estrous

MCA factor map revealed a cluster of variables named ‘positive cluster’. Treatment with retinol, cholecalciferol, and buserelin was grouped with mares that exhibited estrous with serum estrogen level > 40 pg/ml and ovarian follicles size > 30 mm on day 14 of treatment, Fig. 8. The serum progesterone level of mares did not fit into the cluster.

## Discussion

This study aimed to investigate clinical relationship between retinol, cholecalciferol, and buserelin in inducing estrous in late-season anestrous mares. To date, no research has explored effects of administering retinol and cholecalciferol on the effectiveness of buserelin for inducing estrous in anestrous mares. In groups A, B, and C (control group), 71.43%, 28.57%, and 0% of mares exhibited estrous, respectively. The MCA factor map identified a group of variables named ‘positive cluster’. Treatment with retinol, cholecalciferol, and buserelin was grouped with mares that exhibited estrous with serum estrogen level > 40 pg/ml and ovarian follicular size > 30 mm on day 14 of treatment. Mares that responded had an onset of estrous between days 12 and 13 and reached a > 30 mm follicle size on day 14 of the treatment. In a previous experiment, it was observed that mares developed 35 mm follicles after receiving FSH treatment for five to six days (Meyers-Brown et al. 2017). In our study, it required eight doses (day 7–14) of buserelin, a GnRH analogue, to achieve a follicular size exceeding 30 mm. This result may be attributed to the functional differences between a GnRH analogue and FSH, which is not a GnRH analogue but a gonadotropin. Recently, GnRH analogues such as buserelin, deslorelin, and naltrexone have been used successfully to induce estrous and follicular development in mares experiencing late-season anestrous and fall transition (Kwong and Klein 2019; Zafar et al. 2020).

Mares remain in anestrous during the shorter days of the year (Polasek et al. 2017; Zafar et al. 2020). Melatonin, a dark hormone, increases during short days, leading to GnRH downregulation (Dini et al. 2019). When the level of FSH decreases, the growth and development of follicles also decreases. Similarly, a lower level of LH results in the failure to develop dominant follicles in the winter season (Polasek et al. 2017; Zafar et al. 2020). In this report, follicular size of the responsive mares in group A, which exhibited estrous, was > 30 mm on day 14 of treatment. Buserelin may have caused secretion of FSH (Polasek et al. 2017; Zafar et al. 2020), which helped follicular growth. In a previous experiment, it was observed that mares developed 35 mm follicles with FSH treatment (Meyers-Brown et al. 2017). It has been reported that administration of buserelin also induces estrous in bitches during the anestrous phase (Rezende et al. 2018). Previously, buserelin treatment at a dosage of 10.50 μg/day resulted in estrous induction in 33.3% of anestrous mares, while no mare in the control group exhibited estrous (Zafar et al. 2020). Interestingly, when Vitamin E (1.05 g/animal) and selenium (2.25 mg/animal) were added to buserelin treatment, 83.3% of the anestrous mares exhibited estrous (Zafar et al. 2020). This may be because studied mares were deficient in Vitamin E and/or selenium. Administering these nutrients likely addressed this deficiency and improved reproductive performance, similar to previous studies in cattle, sheep (Hemingway 2003), and rabbits (Fadl et al. 2024). However, this demonstrates effective use of GnRH analogues in inducing estrous in anestrous mares.

The ovarian follicles and serum estrogen levels were higher in group A mares, which received retinol and cholecalciferol, compared to group B. The role of retinol and cholecalciferol in estrous induction in mares has not been studied, but their role in follicular development and oocyte maturation is well established (Ikeda et al. 2005; Xu et al. 2021). Cholecalciferol plays a vital role in the development of follicles and ovarian function, from activation of primordial follicles to the production of mature oocytes. It can directly affect follicular cells and oocytes by regulating its downstream factors and cause an indirect effect by mediating actions of gonadotropins (Xu et al. 2021). This may be because cholecalciferol plays a crucial role in regulating blood calcium levels, and calcium is a universal carrier of biological signals that regulate almost every cell function. Therefore, cholecalciferol and calcium work together to optimize female reproductive health and performance (Giorgi et al. 2018; Dickerson et al. 2022; Safari et al. 2022). Moreover, retinol has positive effects on fertility and promotes maturation of oocyte cytoplasm. It also modulates gene expression of gonadotropin receptors in ovarian follicles (Ikeda et al. 2005). This suggests that retinol and cholecalciferol can improve ovarian function and enhance effectiveness of buserelin in anestrous mares. This may be the reason that a higher number of group A mares exhibited estrous compared to group B, and the size of ovarian follicles and serum estrogen levels were greater in mares of group A, which received retinol and cholecalciferol, than in mares of group B. However, further investigations are needed to uncover underlying mechanisms.

In MCA factor map, treatment with retinol, cholecalciferol, and buserelin was grouped with mares having serum estrogen > 40 pg/ml on day 14 of treatment. The increase in serum estrogen is due to higher follicular size in these mares (McCue 2014). This may occur because administering buserelin stimulates release of FSH and LH, promoting follicle growth (Miki et al. 2016). Granulosa cells in developing follicles produce estrogen (McCue 2014). Our study showed mares treated with buserelin, along with retinol and cholecalciferol, and exhibiting signs of estrous had higher serum estrogen levels. Similarly, in previous research, after daily treatment with buserelin (10.50 μg/day) for up to 10 days, estrous mares had serum estrogen levels reaching as high as 43.83 pg/ml (Zafar et al. 2020).

The serum progesterone levels remained similar in all three groups (0.17–0.23 ng/ml). Previously, a comparable level of progesterone (< 1 ng/ml) was found in seasonally anestrous mares (King et al. 1993; Zafar et al. 2020). The decline in progesterone levels in mares during the seasonal anestrous results from decreased LH and prolactin concentrations due to short daylight (Johnson 1986). Moreover, during winter season, cholinergic system in mares may inhibit release of progestins (Kasson and Hsueh 1985). Furthermore, it is important to note that our study has some limitations, particularly the inability to monitor blood levels of retinol and cholecalciferol before and after treatment. It is possible that studied mares were deficient in retinol and cholecalciferol before the start of treatment, and administration of these nutrients might have addressed this deficiency and improved their reproductive performance. Additionally, it remains unclear how retinol and cholecalciferol work together with buserelin to achieve a higher rate of estrous induction in anestrous mares compared to using buserelin alone. Further research is necessary to investigate this approach for improving equine reproduction and to better understand its underlying mechanisms.

## Conclusions

Thoroughbred mares in late-season anestrous during early winter were successfully induced into estrous. The combination of retinol and cholecalciferol with buserelin increased the rate of estrous induction, promoted follicular growth, and elevated serum estrogen levels as compared to treatment with buserelin alone. Further studies are necessary to determine the underlying mechanisms and how retinol and cholecalciferol enhance the effectiveness of buserelin in inducing estrous.

## Data Availability

The raw data supporting this research will be available upon request from the corresponding author.
